# Solar-Powered Direct Air Capture: Techno-Economic and Environmental Assessment

**DOI:** 10.1021/acs.est.3c08269

**Published:** 2024-01-25

**Authors:** Enric Prats-Salvado, Nipun Jagtap, Nathalie Monnerie, Christian Sattler

**Affiliations:** † German Aerospace Center (DLR), Institute of Future Fuels, Linder Höhe, 51147 Cologne, Germany; ‡ RWTH Aachen University, Chair for Solar Fuel Production, Templergraben 55, 52062 Aachen, Germany

**Keywords:** carbon capture, carbon dioxide removal, CCU, CCS, negative emissions technologies, solar energy, life cycle assessment

## Abstract

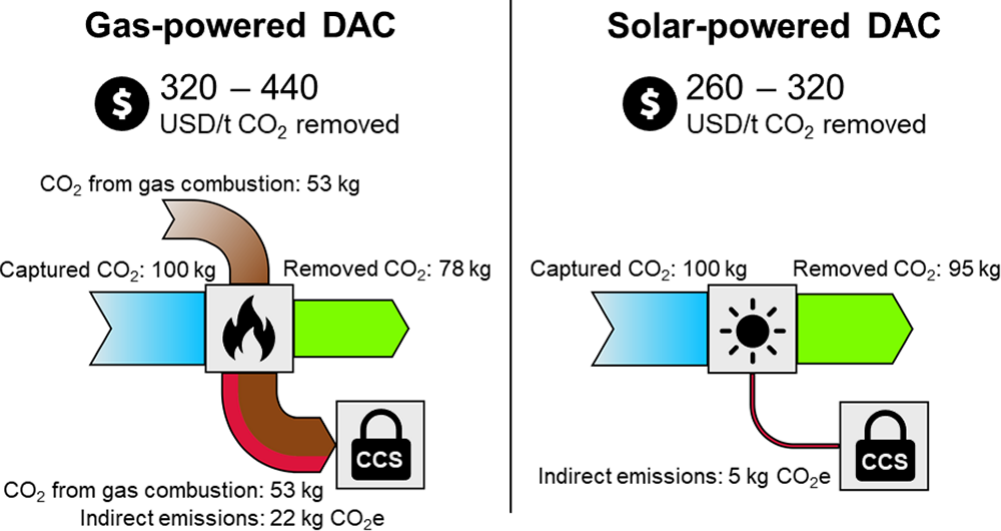

Direct air capture (DAC) of CO_2_ has gained attention as a sustainable carbon source. One of the most promising technologies currently available is liquid solvent DAC (L-DAC), but the significant fraction of fossil CO_2_ in the output stream hinders its utilization in carbon-neutral fuels and chemicals. Fossil CO_2_ is generated and captured during the combustion of fuels to calcine carbonates, which is difficult to decarbonize due to the high temperatures required. Solar thermal energy can provide green high-temperature heat, but it flourishes in arid regions where environmental conditions are typically unfavorable for L-DAC. This study proposes a solar-powered L-DAC approach and develops a model to assess the influence of the location and plant capacity on capture costs. The performed life cycle assessment enables the comparison of technologies based on net CO_2_ removal, demonstrating that solar-powered L-DAC is not only more environmentally friendly but also more cost-effective than conventional L-DAC.

## Abstract 2

Capturing atmospheric CO_2_ with solar energy could be a more efficient, scalable, and cost-effective solution to mitigate climate change.

## Introduction

Direct air capture (DAC) refers to technologies that separate and concentrate atmospheric carbon dioxide solely through mechanical and chemical processes, thus excluding the use of biogenic sources for this purpose.^1−4^ Although biomass-based processes are significantly more mature and less expensive, DAC does not present biophysical limitations that endanger crop production or biodiversity when massively scaled up.^5−9^ CO_2_ from both technologies can be sequestered or used as a feedstock. If a sequestration process is included, the technologies are often referred to as direct air carbon capture and storage (DACCS) and biomass with carbon removal and storage (BiCRS).^10,11^ Since their ultimate goal is to remove carbon dioxide from the atmosphere, they are considered carbon dioxide removal (CDR) technologies, just like nature-based approaches such as enhanced weathering of minerals.^4,12,13^ While CDR is a critical tool to address climate change, atmospheric carbon utilization is also considered a key enabler of the energy transition, as chemicals and fuels derived from nonfossil CO_2_ could be carbon-neutral.^14,15^ These synthetic fuels are considered crucial for decarbonizing hard-to-abate sectors in most energy transition plans.^11,16−18^

The two most mature DAC technologies are solid sorbent DAC (S-DAC) and liquid solvent DAC (L-DAC). On the one hand, S-DAC captures CO_2_ using a solid sorbent, which is later regenerated using vacuum and low-temperature heat (around 100 °C).^3,19,20^ While this process can use low-cost, low-carbon heat sources such as industrial waste heat or geothermal energy, it consumes slightly more energy than L-DAC,^21−23^ and its feasibility demands further development of inexpensive sorbents with higher durability and efficiency.^24−28^ L-DAC, on the other hand, employs a liquid alkali solution that reacts with atmospheric CO_2_ to form carbonates. These carbonates are subsequently calcined to release pure CO_2_. The calcination of calcium carbonate (CaCO_3_), which is commonly preferred in L-DAC, occurs at 900 °C under conventional process conditions.^23,29^ The most advanced L-DAC concepts propose to achieve these high temperatures using oxyfuel combustion of natural gas and capturing the CO_2_ generated.^23^ However, the combustion of natural gas could be considered a suboptimal solution since it contributes up to one-third of the CO_2_ produced in the process.^7,23,30^ Therefore, recent studies have investigated the use of hydrogen^30^ or electricity^23,31^ for calcination. To the best of the authors’ knowledge, the application of solar thermal energy has only been suggested and neither techno-economic nor environmental evaluations were performed.^4,32,33^

Solar thermal energy (STE), particularly solar towers, is a mature yet relatively new technology that can provide renewable energy in the form of heat at temperatures above 1000 °C with a remarkably low carbon footprint.^34−36^ In regions with optimal solar resources the cost of solar heat is also highly competitive.^37−39^ Nonetheless, the use of STE in the calcination unit of L-DAC poses some challenges. First, STE depends on solar irradiance, which is inherently intermittent. Although heat storage is commonly used in commercial STE plants, the high-temperature requirements for L-DAC calcination exclude most of the available storage technologies.^40^ As a consequence, a solarized L-DAC plant must be divided into two distinct sections: continuous and intermittent. The continuous section includes all of the processes that run independently of the STE, as opposed to the intermittent solar calcination, which runs only during the day. Second, even though solar calcination is being actively researched, it has not yet reached commercial maturity. Therefore, the costs and efficiencies can still be substantially improved.^41,42^ Finally, STE and L-DAC respond to environmental conditions in different ways. On the one hand, STE is more economical in locations with strong and stable solar radiation throughout the year. Because of these conditions, arid climates often prevail at these sites. On the other hand, the carbon removal efficiency of L-DAC is generally favored by high humidity, whereas it suffers significant water losses in hot and dry environments.^43^ Furthermore, some of the best countries for STE are classified as developing economies where the ability to raise funds for capital-intensive projects, such as STE or L-DAC plants, is comparatively low. As a result, these locations typically have a higher weighted average cost of capital (WACC), which is detrimental to the levelized cost of removed CO_2_ (LCOD).^44−46^ These conflicting characteristics complicate the search for the most convenient site to place a solar-powered L-DAC plant.

In a nutshell, a proper assessment of solarized L-DAC calls for a methodology that considers both the physical and economic diversity across a wide range of sites as well as the temporal variability of climatic conditions at each of these sites.

## Materials and Methods

The solar-powered L-DAC is a modified version of the process originally published by the Canadian company Carbon Engineering Ltd.^23^ This process captures CO_2_ from the atmosphere in two cycles. In the first cycle, an alkali solution, specifically potassium hydroxide, is introduced to an air contactor, allowing atmospheric CO_2_ to react with the alkali. This reaction yields potassium carbonate, which is fed into a pellet reactor, where it reacts with calcium hydroxide to form calcium carbonate (CaCO_3_) and potassium hydroxide. The potassium hydroxide is then recirculated to the air contactor. In the second cycle, calcium carbonate is calcined to generate pure CO_2_ and calcium oxide (CaO). To complete the cycle, CaO is combined with water in the steam slaker producing calcium hydroxide through a highly exothermic reaction.

**Figure 1:**
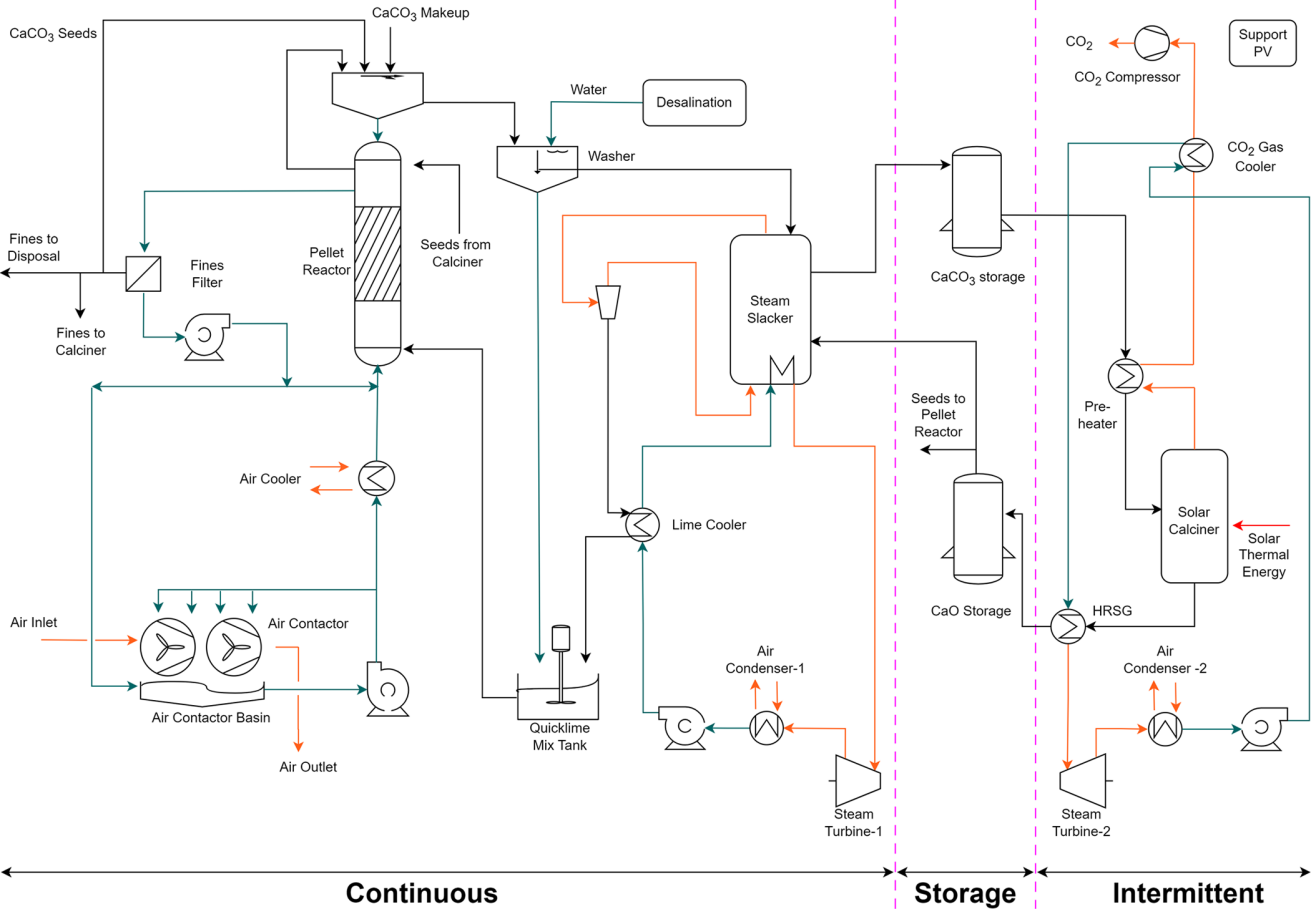
Process flow diagram of solar liquid direct air capture (L-DAC). Stream colors indicate their respective states: orange for gaseous streams, blue for liquid streams, and black for solid streams. Within the solar calciner, the red arrow indicates the input of solar thermal energy. HRSG stands for a heat recovery steam generator.

The primary difference between the conventional and solar-powered L-DAC systems lies in the substitution of the gas-fired calciner with a solar calciner. This also eliminates the need for the air separation unit, the gas turbine for electricity production, and the turbine’s flue gas CO_2_ absorber. While conventional calciners typically have very large capacities, the solar calciner considered in this study is limited to a thermal power of 41.7 MW under design conditions, and only one can be installed in each solar tower.^47,48^ As previously mentioned, the plant is divided into continuous and intermittent sections, as shown in Figure 1. Each of these sections is equipped with a steam turbine (as opposed to a single turbine in the original process) that converts waste heat to electricity, providing an optimal heat recovery strategy. The first steam turbine operates continuously with the heat of the steam slacker, while the second functions in an intermittent regime with waste heat from the CO_2_ gas cooler and the heat recovery steam generator (HRSG). To serve as a buffer between the continuous and intermittent sections, two solid storage units are incorporated into the system. Additionally, the system incorporates water and carbon dioxide storage tanks as well as an auxiliary photovoltaic (PV) plant with batteries that guarantee the stable operation of the plant throughout the year.

This process was simulated in Aspen Plus V12.1 software for steady-state operation. Parameters derived from this model were utilized for sizing and cost estimation of the primary units, assuming a linear relationship with scaling (e.g., if the plant size is doubled, the stream entering the pellet reactor or the turbine output will also double). Further documentation about the process model can be found in the Supporting Information.

### Solar Calcination Modeling

To determine the required size of the solar field, DLR’s HFLCAL VH13 software was employed. This software enables the calculation of the solar field size capable of providing a specified heat at the top of the solar tower for specific locations under design conditions (i.e., clear sky at solar noon on the day of the equinox). It also yields other valuable parameters, such as the optimal tower height and average hourly solar field efficiency. HFLCAL considers factors like tower shading, blocking between heliostats, cosine effect, or mirror absorption.^49^

The selected solar calcination technology is the CentRec receiver, chosen for its simplicity and relatively high technology readiness level (TRL).^47,48^ The heat output of the field was multiplied by the reactor efficiency, which was found to be 87.8% (with an incident flux of 1.7 MW/m^2^) under the design conditions of 900 °C and an aperture diameter of 3 m.^47^ According to our Aspen Plus simulation, the heat requirement of the solar calciner is 1.51 MWh/t CO_2_. Additionally, the cold start-up of the system was accounted for by excluding the irradiation during the first hours of the day. The precise quantity discarded is assumed to be 10% of the average daily heat collected by the solar field.^50^ If the excluded quantity exceeded the total irradiation for that day, then the operation was deemed unfeasible for that specific day.

### Air Contactor Modeling

According to the publications by Carbon Engineering Ltd., the air contactor is a unit composed of fans that propel air horizontally through packing material. This material is continuously moistened by a falling film of alkali solution.^51−53^ In the current study, and based on the aforementioned sources, air velocity and pressure drop within the contactor packing and demisters were assumed to be constant at 1.4 m/s and 100 Pa, respectively. The fan efficiency and the atmospheric CO_2_ concentration were also assumed to be constant at 70%^23^ and 420 ppm,^54^ respectively. To take the environmental conditions into account, data from a pertinent study^43^ was incorporated by fitting it into a polynomial using Python. Details of the fittings can be found in the Supporting Information. This approach enabled the calculation of carbon removal efficiency and water losses as functions of the dry-bulb temperature and relative humidity. Afterward, these equations were used to determine the hourly amount of CO_2_ captured and the associated water losses, facilitating the accurate sizing of the air contactor and the desalination plant, respectively.

### Screening of Locations

Given the high water consumption of L-DAC, the spatial dimension was limited to coastal areas less than 100 km from the ocean, which is assumed to be the practical limit for using desalination as a water source.^55^ Similarly, only latitudes below 45° in both hemispheres were included to guarantee sufficient solar irradiation throughout the year.^37^ These constraints were applied to a world map with a resolution of 110 km, and the obtained area was divided into polygons in the QGIS geographic data processing software. Regions below 1000 km^2^ were discarded and the remaining 672 polygons were divided into 10 × 10 km cells. The land availability of each of these cells was analyzed using three criteria consistent with existing STE potential studies:^38,46^ (1) the maximum slope in the cell must be less than 2.1%,^56^ (2) the current land cover class must be “Shrubs”, “Herbaceous vegetations” or “Bare/sparse vegetation”,^57^ and (3) the cell cannot be part of a protected area.^58^ Cells that violated one or more of these criteria were considered unsuitable and discarded. Finally, the number of suitable cells per polygon was counted and if it was less than five (equivalent to 500 km^2^ of available land), the entire polygon was discarded. This resulted in a total of 282 polygons, which were transformed into representative locations by finding their centroids. The coordinates of each centroid were fed into Meteonorm to obtain the meteorological data for that location.

### Equipment Sizing

To size the equipment, the hourly meteorological data from Meteonorm of each location were fed into the model. This enabled the calculation of annual CO_2_ production for both continuous and intermittent sections, assuming a 90% utilization rate. Continuous production depends on the air contactor and continuous part sizing, while intermittent production is determined by the solar field capacity and intermittent section sizing. The optimization algorithm (minimize from SciPy) enforced equivalent production for both sections and, by including cost data, identified the optimal ratio between solar equipment size and the peak capacity of the intermittent section to minimize total capital expenditure (CAPEX).

Additionally, the model found minimal size requirements for CaO, CaCO_3_, water, and CO_2_ storage. Water storage was designed to accommodate the highest daily demand observed throughout the year, which was also considered to be the desalination plant’s design capacity (with an associated electricity demand of 3.5 kWh/t H_2_O^59^). Although comparatively minimal, the water demand for the cleaning of heliostats was also included.^60^ CO_2_ storage was planned to hold up to 2 weeks of production.

Finally, electricity consumption and production were modeled for each plant section at hourly resolution to identify periods when the amount of electricity generated by the steam turbines was insufficient. This data was fed into the Greenius software to determine the installed PV power and battery capacity capable of sustaining the plant’s production in an autonomous manner. Even though the plant may not be off-grid in practice, a 100% hourly green power matching capacity has been found to be critical to avoid drastic LCA implications.^61,62^ Sample data along a week of operation can be found in the Supporting Information, where it can be observed that most of the electricity produced by the PV is directly consumed to support the CO_2_ compression. The remainder is used to charge the battery module or potentially sold to the grid, although electricity trading is not considered in this study. Two common situations in which the batteries are needed as a backup are during the night in some locations where fan power consumption is high or during the hottest hours of the day when the PV panels overheat and reduce their output.

### Cost Estimation of Solar L-DAC

The capital investment for solar-powered L-DAC was calculated for each location. To estimate equipment costs, relevant correlations for commercially mature equipment were identified in the literature, and a Lang factor of 4 was applied.^63^ This factor multiplies the equipment cost to account for additional expenses such as transportation, installation, and piping and instrumentation.^63^ In cases where these correlations were not available, such as for lower TRL equipment, the seven-tenth rule was employed. This rule uses existing size and field cost data to calculate the field cost of equipment for another specific size.^64^ The TRL category assigned to each unit and the specific equations utilized are available in the Supporting Information.

Due to the diverse publication years and origins of the consulted sources, cost data were harmonized using the Chemical Engineering Plant Cost Index (CEPCI) for July 2022^65^ and the average Euro-to-Dollar exchange rate for 2022. Subsequently, the cost of the captured CO_2_ was obtained in accordance with the methodology of the work published by Carbon Engineering.^23^ The initial step was determining the total CAPEX using the equations outlined below:
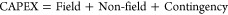
1
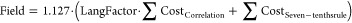
2
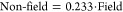
3
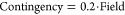
4

Afterward, the fixed operational expenditure (OPEX) was calculated as a function of the CAPEX to account for the maintenance and insurance,^63,66^ while the variable OPEX was calculated by considering the makeup chemicals and labor.^63^ A detailed OPEX calculation methodology, including labor and chemicals estimates, is available in the Supporting Information.
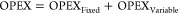
5
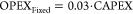
6
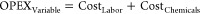
7

Finally, the CAPEX was annualized with the WACC and the expected plant lifetime, which was assumed to be 25 years. The WACC is specific for each country and based on recent literature.^44^ A comprehensive table derived from this source can be found in the Supporting Information. The annualized CAPEX enabled the calculation of the levelized cost of produced CO_2_ (LCOP) and LCOD. In contrast to the LCOP, the LCOD subtracts the indirect greenhouse gas (GHG) emissions associated with the process and the CO_2_ of fossil origin (generated in the conventional process) from the CO_2_ produced. The sequestration of this subtracted fraction is critical to ensure that the final CO_2_ is completely carbon-neutral. Considering the use of onshore storage, the cost of sequestration is assumed to be 10 USD_2022_/t CO_2_.^67^ While the boundary of this study is the delivery of captured CO_2_ at 151 barg to the gate of the plant (thus excluding the cost of transportation to the storage or utilization facilities), we envision the possibility of associating multiple DAC facilities and sequestering the output of the one closest to the sequestration infrastructure to balance the operations of the rest.
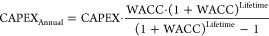
8
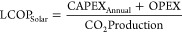
9
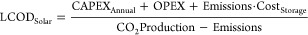
10

To assess the uncertainty associated with the cost estimation, a margin of ±30 and ±50% was considered for high and low TRL equipment, respectively. A Monte Carlo simulation with ten million samples was then used to randomly adjust the costs within the stipulated margins, from which the mean value and the standard deviation were extracted.

### Cost Estimation of Conventional L-DAC

To compare the solar alternative with the conventional one, the field cost of the conventional L-DAC was extracted from the study published by the founders of Carbon Engineering Ltd.^23^ and adjusted to July 2022 using the CEPCI method and the “seven-tenths rule” for different capacities. More information about the exact method of scaling can be found in the Supporting Information. The final CAPEX was calculated from the total field costs as described in eqs 1, 3, and 4. From this, the annualized CAPEX could be obtained as shown in eq 8. The OPEX data were also retrieved from the same source. For the nonenergy-related OPEX, the costs were adjusted with the Consumer Price Index (CPI) for 2022,^68^ while for the energy-related OPEX, the average cost of industrial natural gas in the United States and the European Union was considered in two different scenarios (7.617 USD_2022_/GJ_LHV_^69^ and 23.590 USD_2022_/GJ_LHV_,^70^ respectively). Since the literature reports natural gas requirements (8.81 GJ/t CO_2_) and nonenergy OPEX per ton of CO_2_ captured, these figures had to be recalculated to be expressed per ton of CO_2_ produced using the reported ratio of fossil CO_2_ to atmospheric CO_2_ of 0.48.^23^
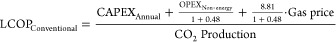
11
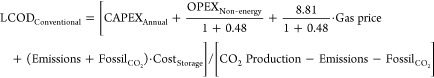
12

### Life Cycle Assessment

Life cycle assessment (LCA) is a methodology that enables the environmental assessment of products or services by collecting all material and energy flows that are exchanged with the environment throughout a life cycle. The environmental impacts of these flows are later quantified and classified into different impact categories, such as climate change or fossil fuel depletion.^71,72^ In the present work, the objective of the LCA is to quantify and compare the GHG emissions associated with captured CO_2_ from cradle-to-gate (i.e., the final application of CO_2_ is explicitly out of scope). System boundaries include, first, the materials required to build the plant, their transportation, and their disposal at the end of the plant’s life and, second, the consumable chemicals and utilities needed. This data was mostly based on an available LCA for L-DAC.^7^

In the present work, a total of four cases were considered: (1) Conventional L-DAC with natural gas from North America, (2) conventional L-DAC with natural gas from Europe, (3) solar-powered L-DAC (Portugal), and (4) solar-powered L-DAC (Chile). As a basis for comparison, the functional unit of “1 t CO_2_ captured from the atmosphere in a plant with a capture capacity of 1 Mt CO_2_/year and a lifetime of 25 years” was utilized. Between cases 3 and 4, the major difference was the size of the solar thermal energy plant. The key impact category assessed to fulfill the LCA’s objective was climate change, but other relevant categories such as metal or fossil fuel depletion were also analyzed. To determine the uncertainty of the LCA, a Monte Carlo simulation was performed with 1000 samples for each case.

The LCA was performed with openLCA 2.0.0 and the EcoInvent 3.7.1 database. The impact assessment method was “ReCiPe Midpoint (H) w/o LT”. A comprehensive table with the inputs and outputs for each case can be found in the Supporting Information.

## Results and Discussion

### Role of Environmental Conditions

The results reveal the effect of local physical and meteorological features on several performance indicators, namely, the electricity required for the fans that pump air in the air contactor, the water losses in the air contactor, and the required size of the solar field for a plant with an annual capacity of 0.1 Mt CO_2_/year (assuming 90% utilization rate).

**Figure 2:**
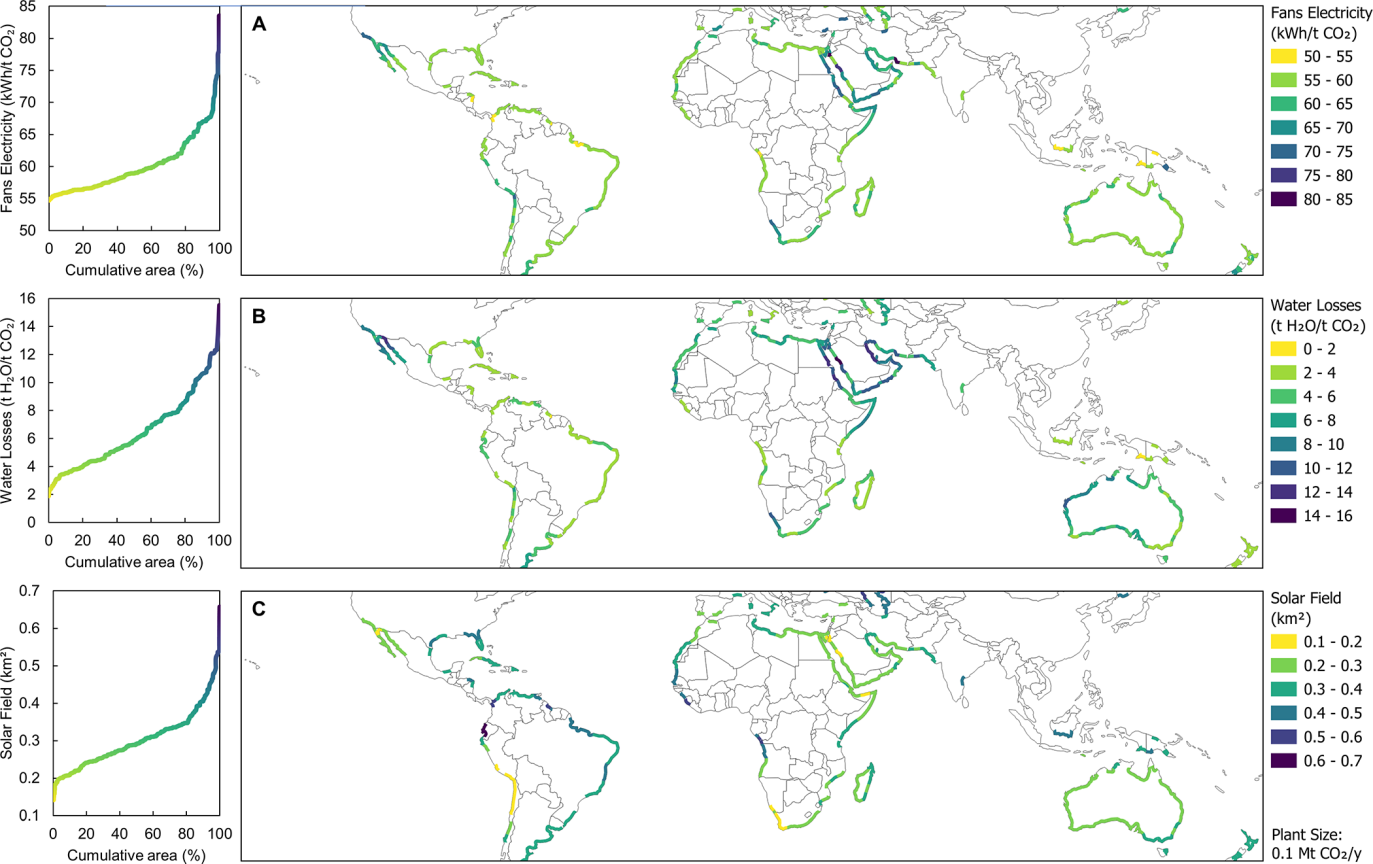
Screening of the impact of environmental conditions on the electricity consumption of fans (A), water losses at the air contactor (B), and solar field area (C).

As visible in the upper plot (A) of Figure 2, specific electricity consumption is generally higher on the western coasts of North and South America or in the Middle East. This phenomenon could be explained by the relatively dry conditions in these areas, which can lead to lower CO_2_ removal efficiency.^43^ Inferior CO_2_ removal efficiency translates to higher CO_2_ concentration at the outlet of the air contactor, and as a result, more air must be processed to achieve equivalent carbon removal. Another important factor is the lower atmospheric pressure at high altitudes, which means that for the same air flow rate, the amount of CO_2_ captured is lower, even if the removal efficiency is high. In some places, such as the Arabian Peninsula and the Peruvian coast, both effects converge, resulting in some of the highest electricity consumption.

The collected data regarding the water intensity of the solarized L-DAC indicate that a third of the screened area has an annual water loss of 4.7 t H_2_O/t CO_2_ or less, which is reported as the expected water loss for the conventional L-DAC.^4,23^ This fact can serve as evidence of the feasibility of operating a solar (or conventional) L-DAC plant in a wide range of sites. The central plot (B) in Figure 2 shows that the coasts of the Red Sea, as well as the Persian and Californian gulfs, are among the most exposed when it comes to water loss. The common factor across these environments is a combination of both high temperature and low relative humidity for long periods along the year, which explains these findings. Surprisingly, water losses are moderate in the Iberian Peninsula and the western coast of South America, although they generally exhibit drought-prone climates. This is presumably due to the comparatively lower average temperature caused by a higher latitude and altitude.

The lower plot (C) in Figure 2 provides an overview of solar thermal energy availability around the world. As expected, regions with high solar irradiation require smaller fields to capture 0.1 Mt CO_2_/year: while only 4% of the examined area requires a solar field smaller than 0.2 km^2^, 60% of the analyzed surface is below the 0.3 km^2^ threshold, providing a reasonable margin of possibilities with good or very good solar resources. These areas are mainly found in the Baja California Peninsula, the Peruvian and Chilean coasts, South Africa, the Middle East, and Australia. Finally, approximately 10% of the land area in this study requires solar fields larger than 0.5 km^2^ (i.e., more than three times larger than the field for the best site). Therefore, these locations can be deemed completely unfavorable for solarized L-DAC.

### Impact of Location

**Figure 3:**
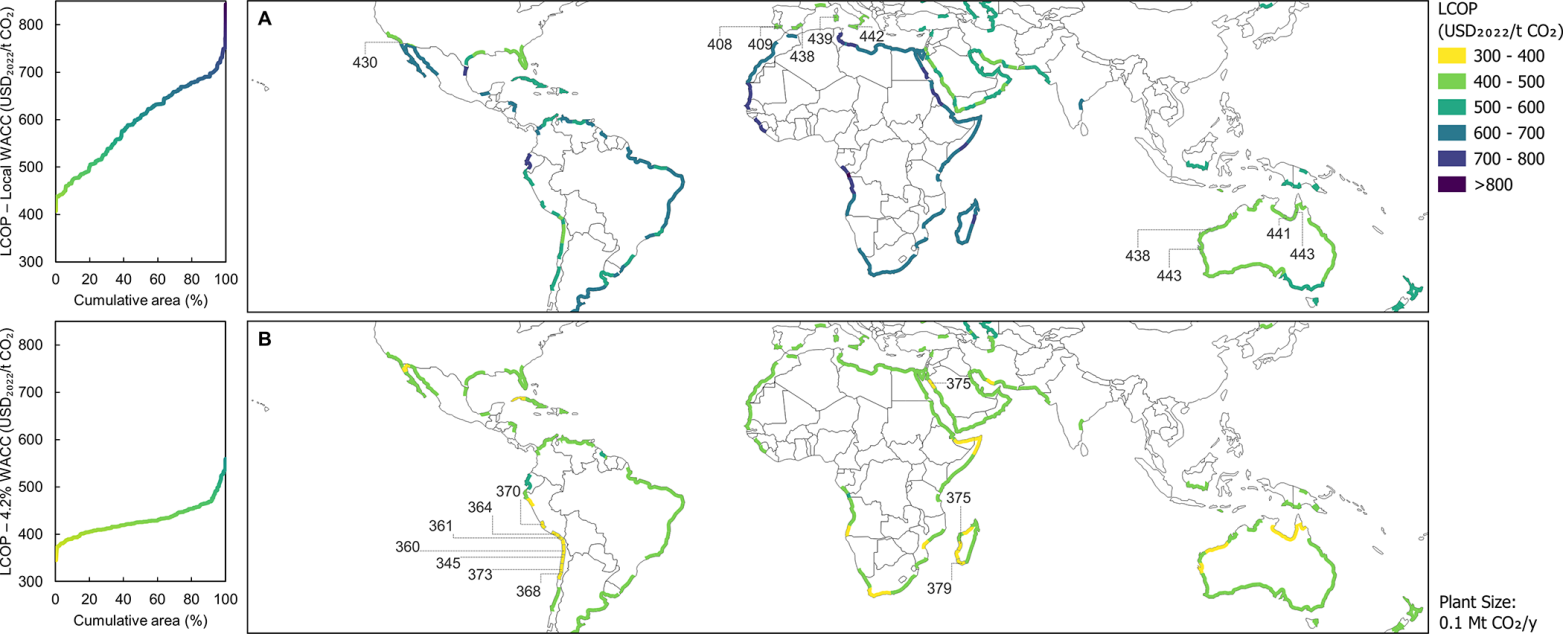
Distribution of levelized cost of produced CO_2_ (LCOP) for a plant size of 0.1 Mt of CO_2_/year around the globe considering (A) local weighted average cost of capital (WACC) and (B) a constant global WACC of 4.2%. In each map, the ten most cost-effective locations are labeled with their LCOP in USD_2022_/t CO_2_.

Based on the aforementioned patterns, the distribution of the levelized cost of produced CO_2_ (LCOP) across the globe shown in Figure 3 can be better understood. It is important to note that these results do not consider the indirect GHG emissions and are valid for only a solar-powered L-DAC plant with a production capacity of 0.1 Mt CO_2_/year and a 90% utilization rate. This plant size was chosen rather than a larger capacity because it is in the same order of magnitude as the maximum annual production of the solar calciner under ideal conditions. This maximum annual production is estimated to be about 0.055 Mt CO_2_/year for a 3 m diameter solar calciner at the best-analyzed location (Chile) with an annual direct normal irradiation of 3.4 MWh/m^2^. This resulted in a total number of solar towers ranging from 2 to 7 across the different locations. Figure 3 contains two subplots: The top (A) displays the LCOP when the local WACC of each site is considered, while the bottom (B) provides an analysis of the LCOP at a constant WACC of 4.2%. This value corresponds to Western European WACC for low-carbon projects^44^ and was arbitrarily chosen in order to assess the suitability of each location from a purely technical perspective. When considering the local WACC, it becomes visible that the most promising sites are in southern Europe, Australia, and California. These findings confirm the great importance of high solar irradiation combined with not-too-harsh environmental conditions that would drastically increase the electricity consumption of the plant as well as the required capacity of the desalination unit. Moreover, these results also show that WACC has a major impact on LCOP, as all of the top 10 sites belong to developed economies with comparatively low WACCs.

The bottom plot (B) of Figure 3 is useful to evaluate the technical potential of each region. By the calculation of the LCOP for all locations with a common WACC of 4.2%, regional socioeconomic differences vanish, and LCOP is influenced only by physical and meteorological constraints. As a result, the top 10 sites have shifted and are now clustered along the Peruvian and Chilean coasts, Southern Africa, and the Middle East. The optimal locations found in South America are consistent with the fact that Chile is one of the STE powerhouses and hosts some of the largest planned and operating projects.^73^ Interestingly, Africa is where the LCOPs drop the most when compared to the top map (A) in Figure 3, due to generally unfavorable WACC.

**Figure 4:**
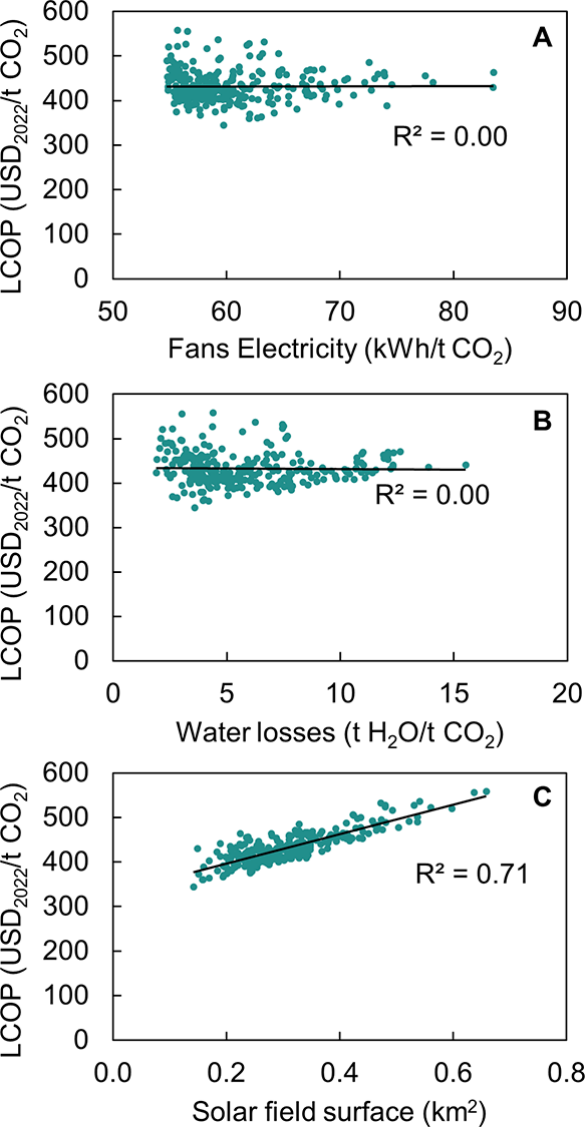
Correlation between the levelized cost of produced CO_2_ (LCOP) for a plant size of 0.1 Mt of CO_2_/year and site-specific environmental conditions, considering a global weighted average cost of capital (WACC) of 4.2%.

Upon integration of the findings presented in Figures 2 and 3, the connection between environmental conditions and the LCOP at a global WACC of 4.2% can be discerned. This relationship is depicted in Figure 4, where it becomes apparent that the LCOP is predominantly influenced by the solar field’s surface area (C). Nevertheless, no significant correlation is detected between the LCOP and either the electricity consumption of the fans (A) or the water losses at the air contactor (B).

### Costs Breakdown

**Figure 5:**
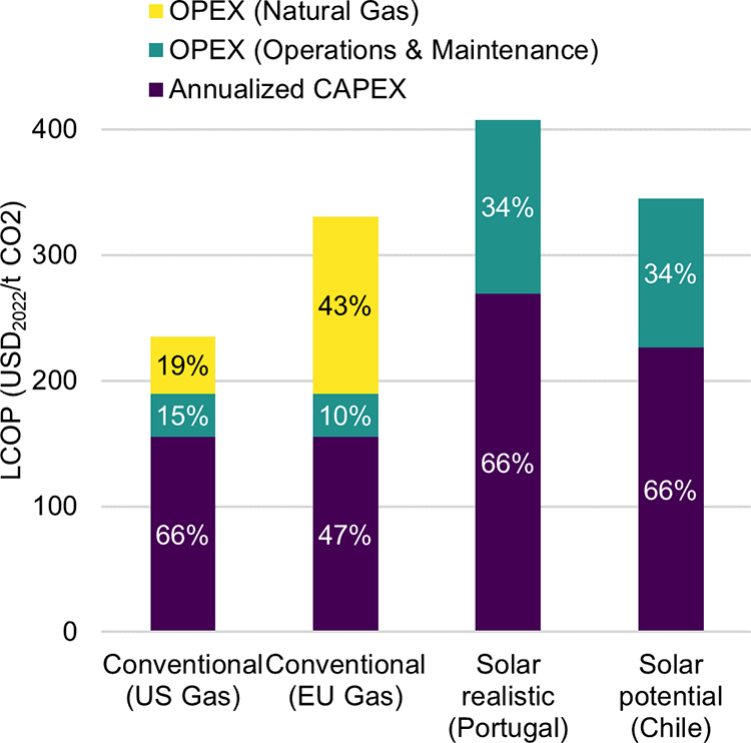
Breakdown of the levelized cost of produced CO_2_ (LCOP) for plants producing 0.1 Mt CO_2_/year. A total of four cases are considered: conventional liquid direct air capture (L-DAC) with United Stated (US) energy costs (i.e., 2022 average cost of natural gas in the United States), conventional L-DAC with European Union (EU) energy costs (i.e., 2022 average cost of natural gas in the European Union), solarized L-DAC in Portugal (most cost-efficient location considering local weighted average cost of capital (WACC)) and solarized L-DAC in Chile (most cost-efficient location considering a global constant WACC of 4.2%).

The LCOP depends not only on the WACC but also on CAPEX and OPEX. A breakdown of LCOP can be observed in Figure 5. To illustrate the influence of energy expenses on the CO_2_ costs for the conventional L-DAC, the OPEX was split into two categories: natural gas and operations and maintenance (which includes both fixed and variable OPEX). As aforementioned, average 2022 natural gas prices for the United States and the European Union were considered to quantify the sensitivity of LCOP to energy price fluctuations. As expected, the contribution of CAPEX to the LCOP is higher for the solarized cases due to their comparatively larger initial investment. It is also noteworthy that in a conservative energy price scenario, natural gas accounts for more than 40% of the LCOP.

**Figure 6:**
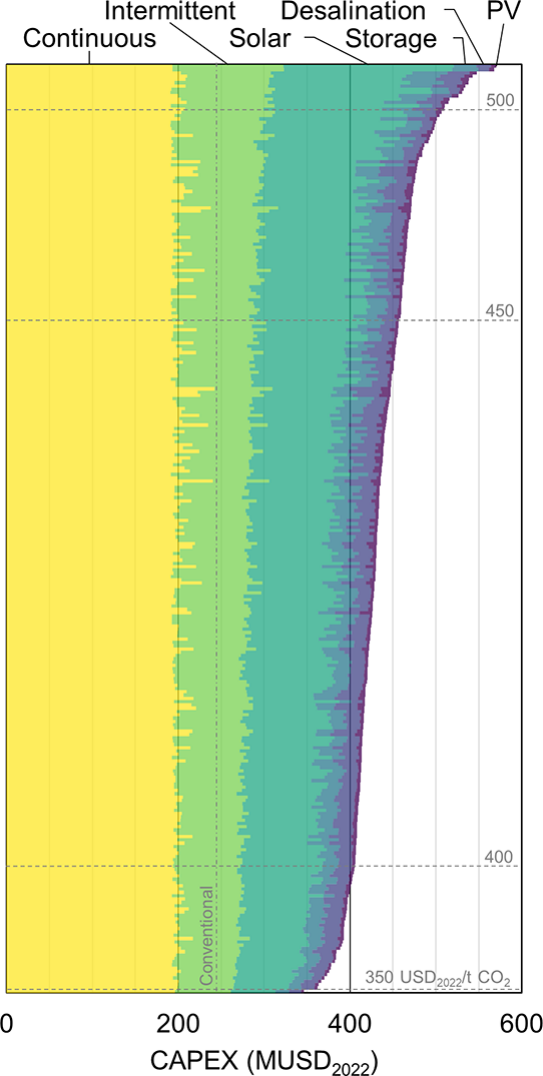
Breakdown of the capital expenditure (CAPEX) of a solarized liquid direct air capture (L-DAC) plant producing 0.1 Mt CO_2_/year for each of the locations analyzed, sorted by the total CAPEX. The levelized cost of produced CO_2_ (LCOP) thresholds of 350, 400, 450, and 500 USD_2022_/t CO_2_ are shown as dashed horizontal lines for reference. Similarly, the CAPEX of a conventional L-DAC plant of equivalent CO_2_ production capacity is shown as a vertical dashed-dotted line for comparison. For more details on the individual components listed under each category, see the Supporting Information.

Interestingly, Figure 5 also indicates that CAPEX for solarized L-DAC varies significantly from site to site. To better understand this, Figure 6 zooms in on the cost breakdown of the CAPEX for each of the analyzed locations sorted by total CAPEX. The key findings are, on the one hand, that the cost of the solar equipment (i.e., heliostat field, solar tower, and solar calciner combined) tends to represent a larger share of the total cost at locations with higher CAPEX, while the other categories tend to remain constant. On the other hand, the share of PV and desalination varies substantially from site to site but remains comparatively small. This implies that these investments are not the main driver for increasing LCOP, but their influence should not be neglected. This last observation can be illustrated by some outliers with low solar CAPEX, but high total investment due to very unfavorable environmental conditions. A more detailed breakdown of CAPEX for both solar and conventional L-DAC can be found in the Supporting Information.

### Impact of Scale

Increasing the amount of CO_2_ captured per year generally has a positive effect on the LCOP, as higher capacities reduce the specific CAPEX (i.e., CAPEX per ton of produced CO_2_). This behavior can also be observed for many other processes due to the economy of scale. Even though the capacity used in the location screening (0.1 Mt CO_2_/year) is 2 orders of magnitude higher than the largest operating DAC plant in the world (4000 t CO_2_/year),^4^ a conventional L-DAC plant with 0.5 Mt CO_2_/year capacity is currently under construction. According to the investors, many more plants with a capacity of up to 1 Mt CO_2_/year will be commissioned in the following decades.^74^ For this reason, the effects of scaling-up in both conventional and solar-powered L-DAC were investigated and are summarized in the left plot (A) of Figure 8.

The results reveal that both conventional and solarized versions of L-DAC experience an improvement in their LCOPs, which is particularly strong in the range between 0.05 and 0.2 Mt CO_2_/year. Also, in this region of the plot, the solarized L-DAC shows a sawtooth pattern due to the impact on the cost of additional solar towers to accommodate the increasing capacity. Interestingly, the pattern vanishes at larger scales as the cost of adding another tower becomes relatively less important. Finally, the results show that the LCOP is clearly lower for the conventional L-DAC than for the realistic solarized process for all capacities studied, although the solar potential case may beat the conventional L-DAC when powered with expensive energy. We conclude that in order to make the LCOP of the solar L-DAC competitive, its CAPEX should be reduced through technological improvements of the solar equipment. However, as shown in the last section, this tendency drastically changes when considering the associated emissions.

### Associated Emissions

Due to the gas-fired calciner used in conventional L-DAC, there is a clear source of fossil CO_2_ that is correctly identified and accounted for in the literature, as the cost is typically reported per ton of CO_2_ removed from the atmosphere (LCOD) rather than per ton of CO_2_ produced (LCOP).^3,4,23^ However, the available techno-economic assessments of conventional L-DAC do not consider the compensation of indirect GHG emissions, which have already been identified in several environmental assessments.^7,75−77^ This simplification may have led to more optimistic LCODs for conventional L-DAC. Similarly, it is of great importance to quantify the indirect emissions of solar-powered L-DAC in order to compensate for them.

**Figure 7:**
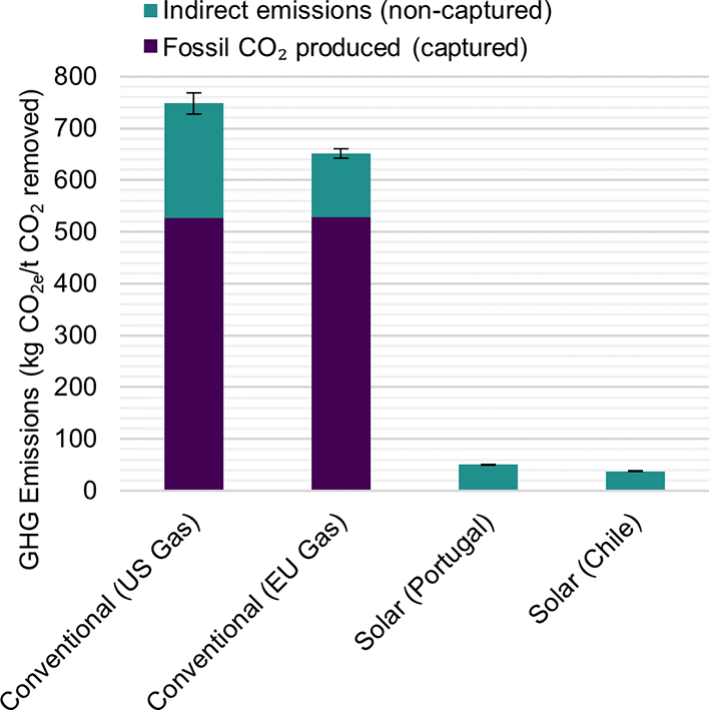
Produced and captured fossil CO_2_ and indirect greenhouse gas (GHG) emissions associated with conventional and solar liquid direct air capture (L-DAC), derived from the climate change impact category of the life cycle assessment. The terms “US” and “EU” stand for United States and European Union, respectively.

Figure 7 presents the LCA results, which are generally consistent with those from previous studies. The results indicate that the indirect emissions for the conventional L-DAC are definitely not negligible. The majority of these indirect emissions stem from the extraction and transportation of natural gas. In the case of the solar scenarios, the primary contributor to indirect emissions is the construction of the solar thermal energy infrastructure. Other impact categories and a breakdown of contributions can be found in the Supporting Information.

### Comparing LCOP to LCOD

**Figure 8:**
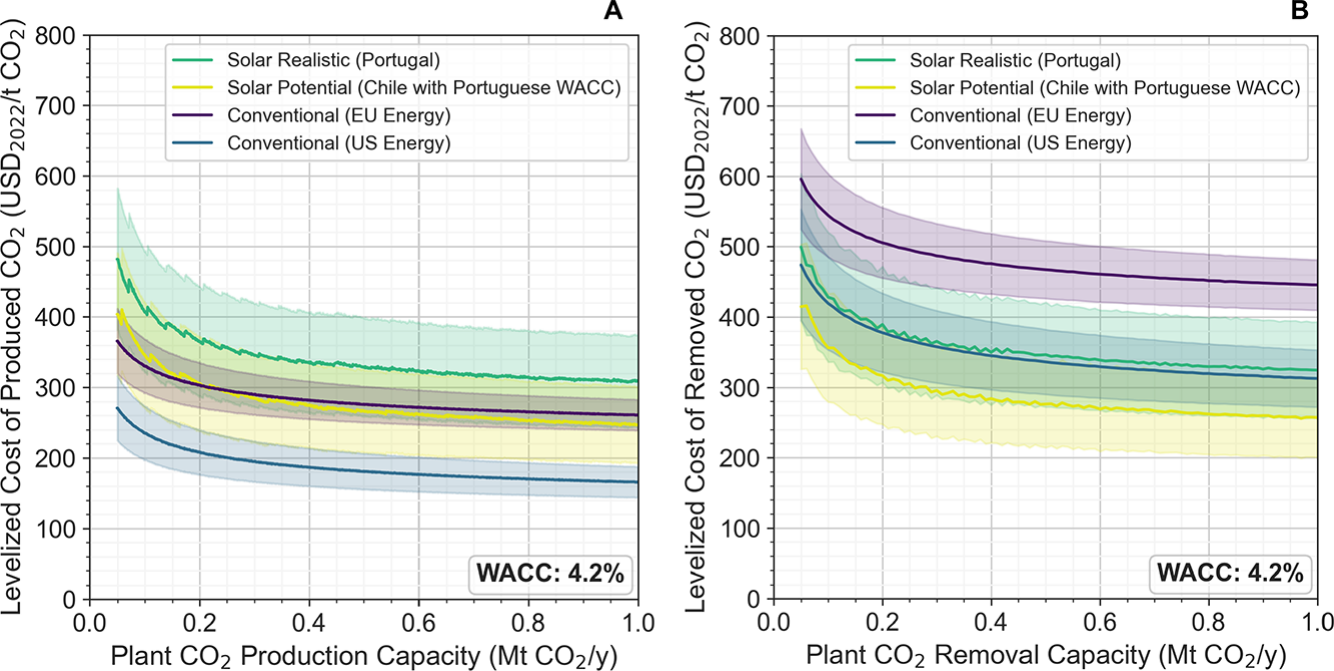
Variation in the levelized cost of produced CO_2_ (LCOP) as a function of the plant’s CO_2_ production capacity (A) and levelized cost of removed CO_2_ (LCOD) in relation to the plant’s CO_2_ removal capacity (B). The terms “WACC”, “US”, and “EU” stand for the weighted average cost of capital, the United States, and European Union, respectively.

As previously introduced, the LCOD, or levelized cost of removed CO_2_, was calculated by subtracting fossil CO_2_ and indirect GHG emissions from the total produced CO_2_ and accounting for additional sequestration costs. Both the uncertainties of the techno-economic assessment and the LCA were considered when generating the results, which can be observed in the right plot (B) in Figure 8. Due to the notably high indirect emissions and fossil CO_2_ generation of the conventional L-DAC technology, its cost escalates dramatically and the realistic solar-powered approach becomes equivalent to the conventional L-DAC powered by low-cost energy. Moreover, the potential solar-powered case becomes the most cost-effective one for all of the analyzed capacities. Although the solarized L-DAC also experiences an increase in the LCOD, the impact is considerably smaller, as the amount of CO_2_ to be offset is more than 1 order of magnitude lower, as observed in Figure 7.

Even though the solar-powered L-DAC has a higher CAPEX than its conventional counterpart, it offers a valuable opportunity to fully decouple a key enabler of the energy transition from fossil fuels. Based on these results, solar thermal energy proves to be a promising alternative for decarbonizing L-DAC, which remains its biggest challenge when competing with other DAC technologies.

## Supporting Information Available

Aspen Plus model summary; Aspen Plus stream summary; CAPEX breakdown; LCA climate change breakdown; LCA impact categories; LCA methodology; result screening; results size Chile; results size Portugal; sample week; Aspen Plus process flow diagram, meteorological maps, land availability, data fitting, cost estimation methodology for solar L-DAC and Cost estimation methodology for conventional L-DAC; and WACC table (ZIP)

## Abbreviations

BiCRS: biomass with carbon removal and storage
CAPEX: capital expenditure
CDR: carbon dioxide removal
CEPCI: Chemical Engineering Plant Cost Index
DAC: direct air capture
DACCS: direct air carbon capture and storage
DLR: German Aerospace Center
EU: European Union
HRSG: heat recovery steam generator
LCA: life cycle assessment
LCOD: levelized cost of removed CO_2_
LCOP: levelized cost of produced CO_2_
L-DAC: liquid direct air capture
LHV: lower heating value
OPEX: operational expenditure
PV: photovoltaics
S-DAC: solid direct air capture
STE: solar thermal energy
US: United States
WACC: weighted average cost of capital
