# Supplementary material for: Solar-Powered Direct Air Capture: Techno-Economic and Environmental Assessment
Source: Environ Sci Technol. 2024 Jan 25;58(5):2282–92. doi: 10.1021/acs.est.3c08269 (PMC10851427; doi:10.1021/acs.est.3c08269)
Supplement: Supplementary file 1 — es3c08269_si_001.zip [file es3c08269_si_001.zip › Supporting_Information.docx]

## SUPPORTING INFORMATION

**Solar-Powered Direct Air Capture: Techno-Economic and Environmental Assessment**

Enric Prats-Salvado^1,2,3,*^, Nipun Jagtap^1^, Nathalie Monnerie^1^ and Christian Sattler^1,2^

^1^German Aerospace Center (DLR), Institute of Future Fuels, Linder Höhe, 51147 Cologne, Germany

^2^RWTH Aachen University, Chair for Solar Fuel Production, Templergraben 55, 52062 Aachen, Germany

^3^Lead contact

*Corresponding author: [enric.pratssalvado@dlr.de](mailto:enric.pratssalvado@dlr.de)

17 pages (including cover) containing 8 figures and 3 tables

## Aspen Plus Process Flow Diagram


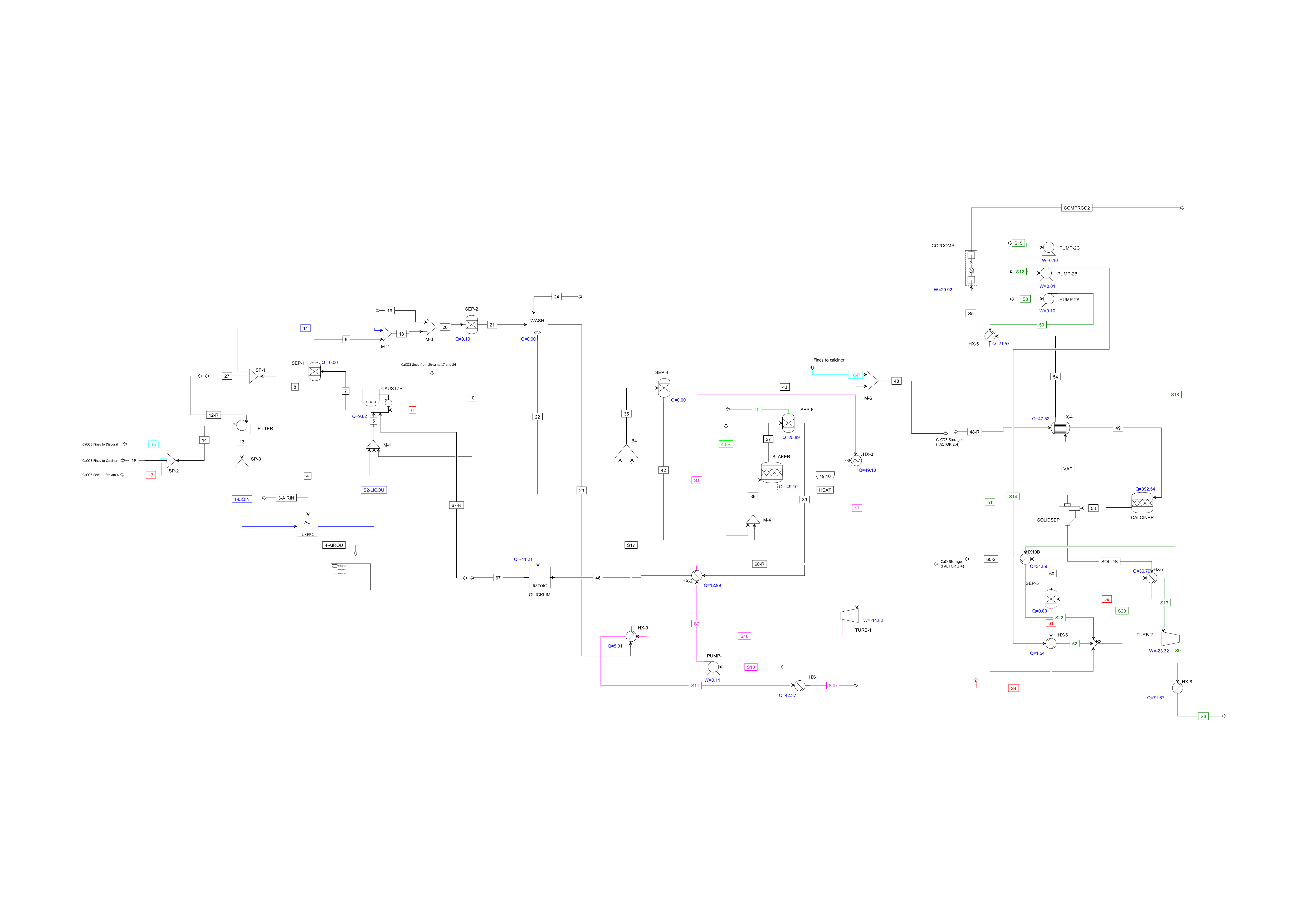


**Figure S1.** Process Flow Diagram (PFD) used in Aspen Plus to simulate the solar L-DAC process. Colors have been arbitrarily assigned to different streams for ease of identification.

## Meteorological maps


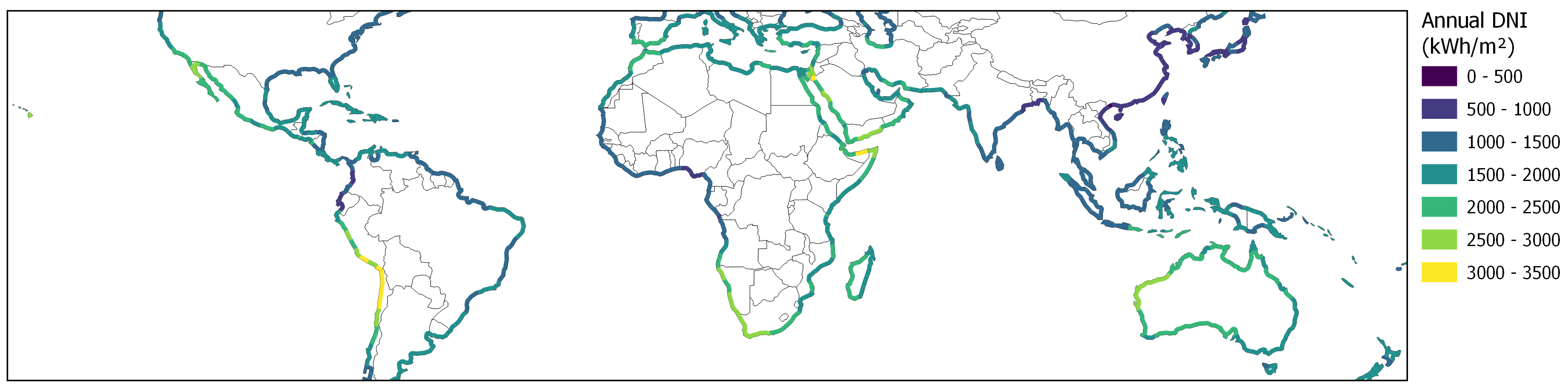


**Figure S2.** Global annual DNI (kWh/m^2^).


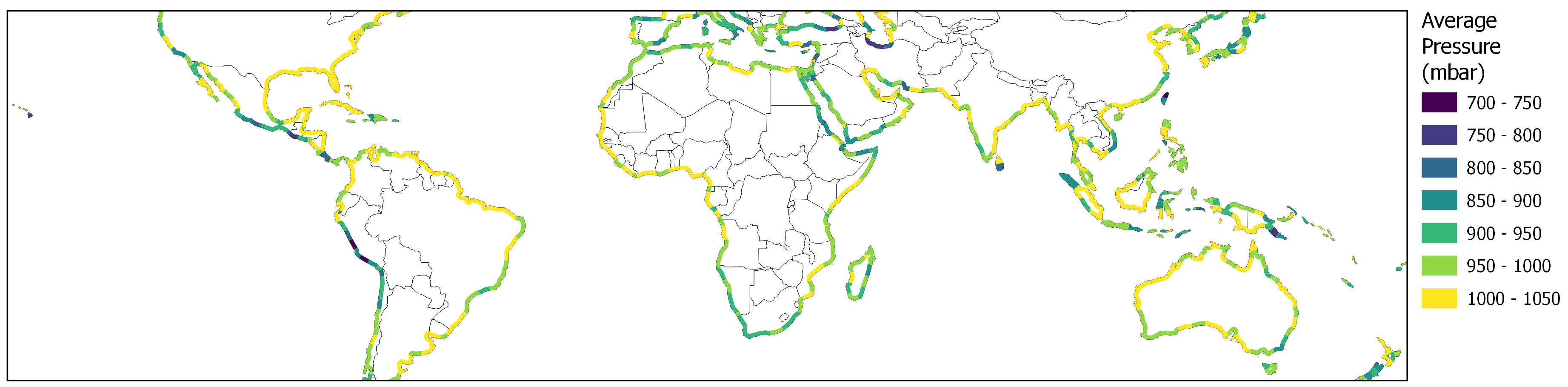


**Figure S3.** Global average pressure (mbar).


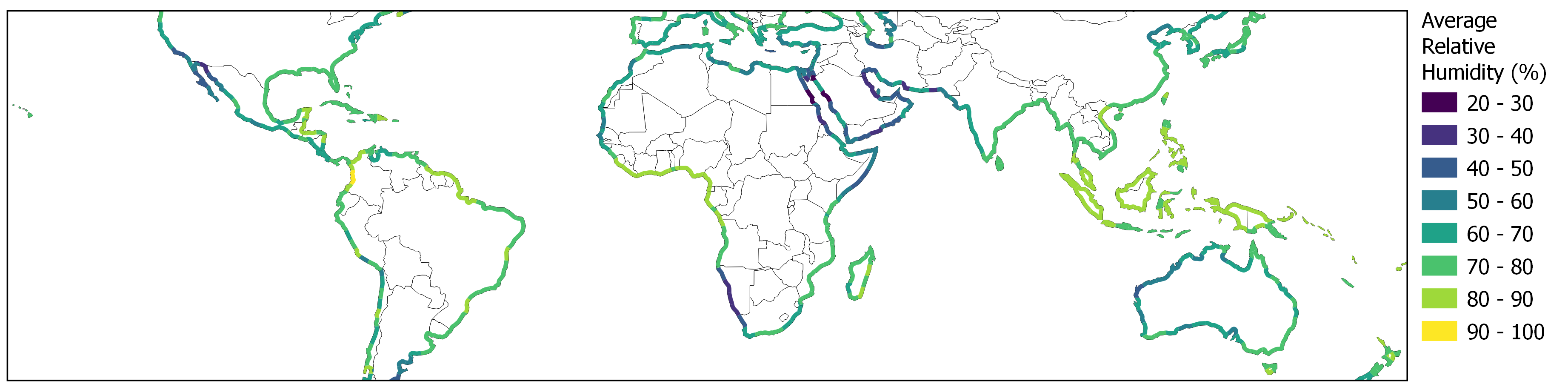


**Figure S4.** Global average relative humidity (%).


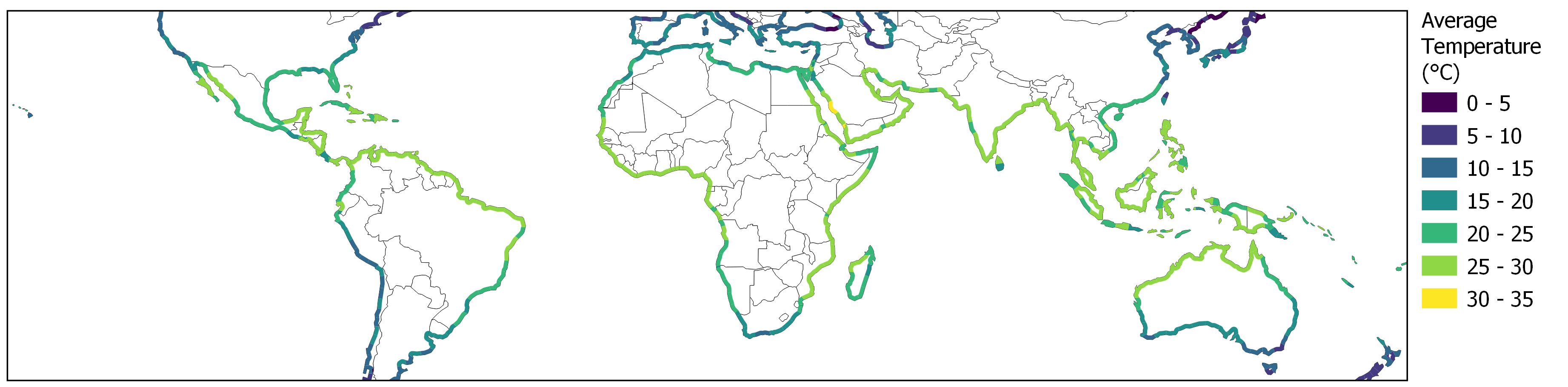


**Figure S5.** Global average temperature (°C).

## Land availability


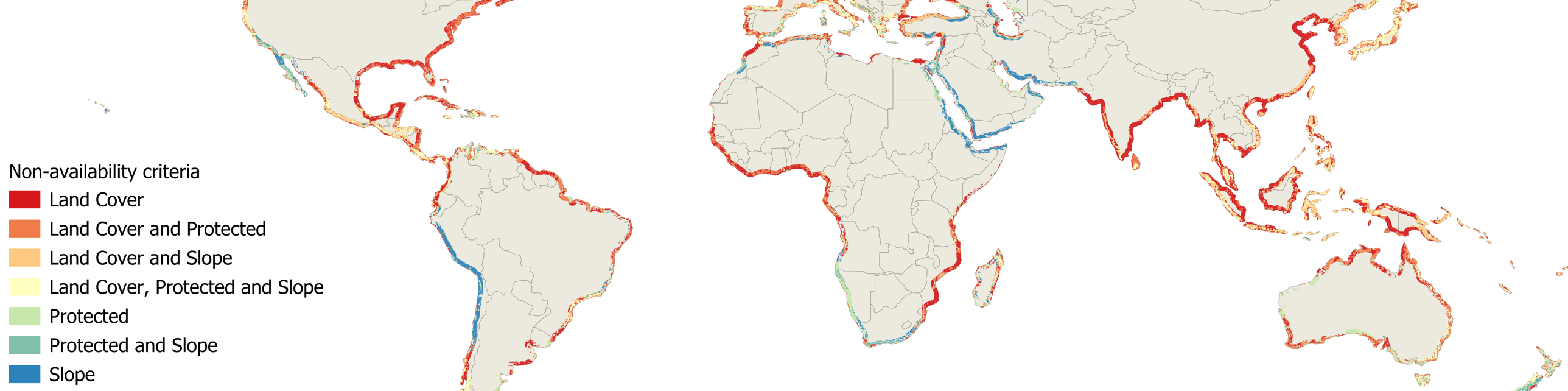


**Figure S6.** Non-availability criteria met for unavailable land.


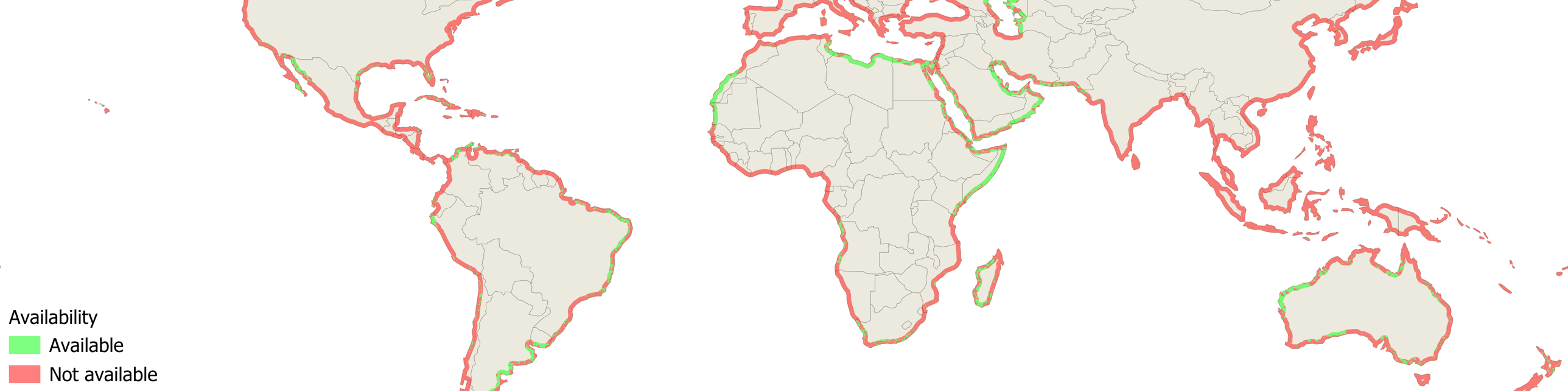


**Figure S7.** Availability of land (a cell is considered available if all three non-availability criteria are respected).


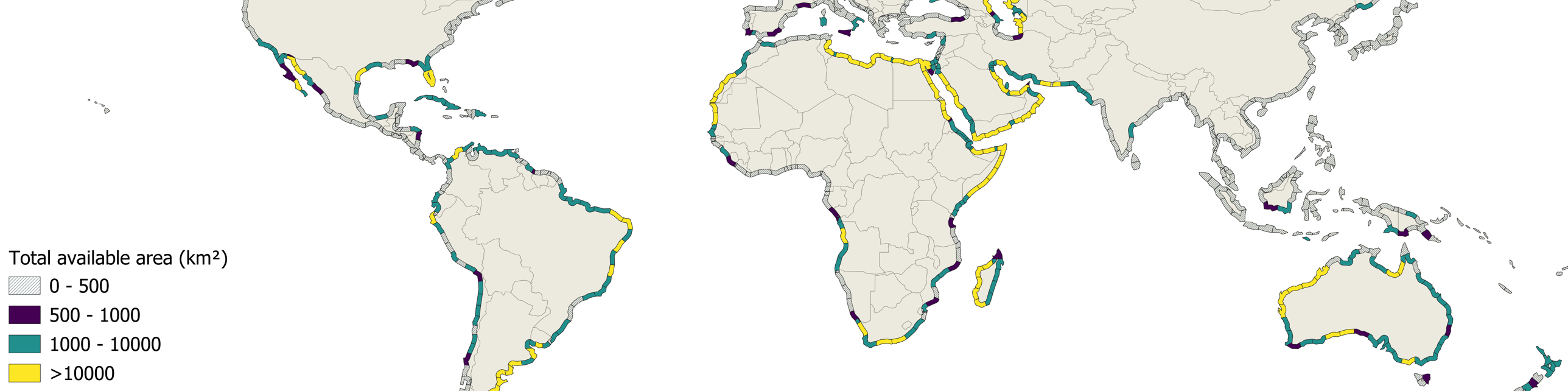


**Figure S8.** Total area available at each site. Sites containing less than 500 km^2^ of available land were discarded and not analyzed.

## Data fitting

| $y=a\cdot T^{2}+b\cdot RH^{2}+c\cdot T\cdot RH+d\cdot T+e\cdot RH+f$ | Equation 1 |
| --- | --- |

**Table S1.** Parameters obtained from the data fitting of carbon removal efficiency and water losses. The original data was extracted from the literature^1^.

| Parameter | Carbon Removal Efficiency (%) | Water Losses (t H_2_O/t CO_2_) |
| --- | --- | --- |
| a | -5.863∙10^-4^ | -2.254∙10^-3^ |
| b | -5.990∙10^-3^ | 7.189∙10^-4^ |
| c | 2.675∙10^-3^ | -5.376∙10^-3^ |
| d | 1.437∙10^-1^ | 5.105∙10^-1^ |
| e | 9.701∙10^-1^ | -1.551∙10^-1^ |
| f | 4.876∙10^1^ | 9.661 |

## Cost estimation methodology for Solar L-DAC

The results of the Aspen Plus^®^ simulation were used to estimate the cost of the equipment required for the solar plant. The simulation was run for a specific plant capacity, namely 108.559 t CO_2_/h for the continuous section and 259.974 t CO_2_/h for the intermittent section.

Several variables were derived from the simulation, such as the solution flow rate entering the filter, the CaO flow rate entering the steam slaker or the design power input of CO_2_ compressor. These variables have in common that they are linearly related to the CO_2_ output of the plant (i.e., if the plant capacity is halved, the variables are also halved).

For the sake of simplicity, two factors are defined to simplify the further calculation of costs for each unit. The continuous scale factor (CSF) is a ratio between the CO_2_ design capacity of the continuous section for the studied case and the CO_2_ capacity of this section in the Aspen Plus^®^ simulation:

| $Continuous Scale factor (CSF)=\frac{Continuous CO_{2} flowrate (t {CO}_{2}/h)}{108.559 t {CO}_{2}/h}$ | Equation 2 |
| --- | --- |

Analogously, the intermittent scale factor (ISF) shows the ratio between the CO_2_ design capacity of the intermittent section for the present case and the CO_2_ capacity of the same section in the Aspen Plus^®^ simulation:

| $Intermittent Scale factor (ISF)=\frac{Intermittent CO_{2} flowrate (t {CO}_{2}/h)}{259.974 t {CO}_{2}/h}$ | Equation 3 |
| --- | --- |

To ensure price comparability, all calculated costs were transferred to July 2022 using the Chemical Engineering Plant Cost Index (CEPCI). The July CEPCI was used as the reference one for all years.

Finally, a certain uncertainty was considered for each unit depending on the TRL (30% for high TRL, 50% for low TRL). This uncertainty is used in the Monte Carlo simulation, which randomly generates a variable (called "MC" in the equations) between -1 and 1.

Table S2 provides a summary of the methods used to determine the cost of each unit of the solar system.

1. **Continuous equipment**
   1. **Air contactor**

The seven-tenths power rule is used with a linear scaling factor (i.e. 1) for the air contactor. This unit does not use the CSF since the design flowrate changes depending on the average meteorological conditions of each location.

1. Reference air flow rate^2^: $\mathrm{Re}f_{\mathrm{AF}}$ = $2.11\cdot{10}^{8}$ m^3^/h
2. Reference Air contactor cost^2^: $\mathrm{Re}f_{\mathrm{ACC}}$ = $212.2 \cdot{10}^{6}$ USD_2016_
3. Uncertainty = 50%

| $Cost_{AC}=\left( \left( \frac{Air flow rate}{Ref_{AF}} \right)^{1}\cdot Ref_{ACC} \right)\cdot\frac{{CEPCI}_{2022}}{{CEPCI}_{2016}}\cdot(1+MC\cdot Uncertainty)$ | Equation 4 |
| --- | --- |

- 1. **Pellet reactor**

For the pellet reactor, the seven-tenths power rule is used with 0.675 scaling factor^3^, which is an average index for reactors.

1. Reference cost^2^: $\mathrm{Re}f_{\mathrm{PC}}$ = $130.7 \cdot{10}^{6}$ USD_2016_
2. Uncertainty = 50%
3. Factor (f) = 0.675

| $Cost_{PR}=\left( {CSF}^{f}\cdot Ref_{PC} \right)\cdot\frac{{CEPCI}_{2022}}{{CEPCI}_{2016}}\cdot(1+MC\cdot Uncertainty)$ | Equation 5 |
| --- | --- |

- 1. **Filter**

For the filter, the seven-tenths power rule is used with 0.625 scaling factor^3^, which is an average index for filters.

1. Reference cost^2^: $\mathrm{Re}f_{\mathrm{FC}}$ = $30.9 \cdot{10}^{6}$ USD_2016_
2. Uncertainty = 30%
3. Factor (f) = 0.625

| $Cost_{Filter}=\left( \left( CSF \right)^{f}\cdot Ref_{FC} \right)\cdot\frac{{CEPCI}_{2022}}{{CEPCI}_{2016}}\cdot(1+MC\cdot Uncertainty)$ | Equation 6 |
| --- | --- |

- 1. **Slaker**

For the slaker, the seven-tenths power rule is used with 0.675 scaling factor^3^, which is the average index for reactors. In the checked source, the reference cost is combined with the calciner, but given that the solar system does not have a conventional calciner, it had to be adapted. To do so, the fraction of the reference cost that can be attributed to the slaker was retrieved from relevant literature^4^.

1. Reference cost^2^: $\mathrm{Re}f_{\mathrm{SC}}$= $38.85 \cdot{10}^{6}$ USD_2016_
2. Slaker cost fraction (CF_S_) = 28.6%
3. Uncertainty = 50%
4. Factor (f) = 0.675

| $\mathrm{Cos}t_{\mathrm{Slaker}}=\left( \mathrm{CSF}^{f}\cdot Ref_{\mathrm{SC}}\cdot\mathrm{CF}_{S} \right)\cdot\frac{\mathrm{CEPCI}_{2022}}{\mathrm{CEPCI}_{2016}}\cdot(1+MC\cdot Uncertainty)$ | Equation 7 |
| --- | --- |

- 1. **Continuous turbine**

A correlation is used for the cost estimation of the continuous turbine and the reference power is obtained from the simulation. The correlation is available in the literature as a graph that has been digitalized and fitted to an exponential equation. In order to avoid exceeding the limits of correlation, an upper limit has been set. If reached, a parallel unit is added to reduce the size of the system. This operation is expressed as “Roundup”. To obtain the field cost of the turbine, the Lang factor of 4 is applied, which accounts for the installation of the equipment on site^5^.

1. Reference power continuous turbine: $\mathrm{Powe}r_{\mathrm{ref}_{C}}$ = 14.824 MW
2. Power = $\mathrm{Powe}r_{\mathrm{ref}_{C}}$ $\cdot$ CSF $\cdot$ 1000
3. Units = Roundup (Power / Upper limit)
4. Power_unit = Power / Units
5. Data fitting constant^6^: K = 1240.4
6. Data fitting exponent^6^: α = 0.916
7. Uncertainty = 30%
8. Upper limit^6^ = 5971.6 kW

| $\mathrm{Cost}_{\mathrm{Turbin}e_{C}}=\left( (K\cdot{\mathrm{Power}_{\mathrm{Unit}}}^{\alpha} \right)\cdot Units)\cdot\frac{\mathrm{CEPCI}_{2022}}{\mathrm{CEPCI}_{2002}}\cdot\left( 1+MC\cdot Uncertainty \right)\cdot Lang\_F$ | Equation 8 |
| --- | --- |

- 1. **Other continuous equipment**

Other continuous equipment considers unlisted units such as heat exchangers, pumps, separators and mixers. They are assumed to cost 20%^2^ of the process equipment of the continuous section.

| $Other_{Cont}=(Cost_{AC}+Cost_{PR}+Cost_{Filter}+Cost_{Slaker}+Cost_{{Turbine}_{C}})\cdot0.2$ | Equation 9 |
| --- | --- |

1. **Intermittent Equipment**
   1. **CO_2_ compressor**

A correlation is used for the cost estimation of the CO_2_ compressor and the reference power is obtained from the simulation. The correlation is available in the literature as a graph that has been digitalized and fitted to an exponential equation. In order to avoid exceeding the limits of correlation, an upper limit has been set. If reached, a parallel unit is added to reduce the size of the system. This operation is expressed as “Roundup”. To obtain the field cost of the compressor, the Lang factor of 4 is applied, which accounts for the installation of the equipment on site^5^.

1. Reference power requirement: Power_ref_ = 8.626 MW
2. Power = Power_ref_ ∙ ISF ∙ 1000
3. Units = Roundup (Power / Upper limit)
4. Power_unit = Power / Units
5. Uncertainty = 30%
6. Data fitting constant^6^: K = 3137.6
7. Data fitting exponent^6^: α = 0.675
8. Upper limit^6^ = 5966.9 kW

| ${Cost}_{comp}=\left( (K\cdot{Power_{unit}}^{\alpha} \right)\cdot Units)\cdot\frac{{CEPCI}_{2022}}{{CEPCI}_{2002}}\cdot\left( 1+MC\cdot Uncertainty \right)\cdot Lang\_F$ | Equation 10 |
| --- | --- |

- 1. **Intermittent turbine**

A correlation is used for the cost estimation of the intermittent turbine (equivalent to the continuous turbine) and the reference power is obtained from the simulation. The correlation is available in the literature as a graph that has been digitalized and fitted to an exponential equation. In order to avoid exceeding the limits of correlation, an upper limit has been set. If reached, a parallel unit is added to reduce the size of the system. This operation is expressed as “Roundup”. To obtain the field cost of the turbine, the Lang factor of 4 is applied, which accounts for the installation of the equipment on site^5^.

1. Reference power intermittent turbine: $\mathrm{Powe}r_{\mathrm{ref}_{I}}$ = 23.316 MW
2. Power = $\mathrm{Powe}r_{\mathrm{ref}_{I}}$ $\cdot$ ISF $\cdot$ 1000
3. Units = Roundup (Power / Upper limit)
4. Power_unit = Power / Units
5. Data fitting constant^6^: K = 1240.4
6. Data fitting exponent^6^: α = 0.916
7. Uncertainty = 30%
8. Upper limit^6^ = 5971.6 kW

| $\mathrm{Cost}_{\mathrm{Turbin}e_{I}}=\left( (K\cdot{\mathrm{Power}_{\mathrm{Unit}}}^{\alpha} \right)\cdot Units)\cdot\frac{\mathrm{CEPCI}_{2022}}{\mathrm{CEPCI}_{2002}}\cdot\left( 1+MC\cdot Uncertainty \right)\cdot Lang\_F$ | Equation 11 |
| --- | --- |

- 1. **Other intermittent equipment**

Other intermittent equipment considers unlisted units such as heat exchangers, pumps, separators and mixers. They are assumed to cost 20%^2^ of the process equipment of the intermittent section.

| $Other_{Int}=(Cost_{TurbineI}+Cost_{comp})\cdot0.2$ | Equation 12 |
| --- | --- |

1. **Solar Equipment**

Correlations from relevant literature are used for each solar equipment type.

1. **Heliostat**

The cost of land is also included in the heliostat field by calculating the total land required for the heliostat surface with the field density, which was obtained from our own calculations. The land price was increased by 50% over the original source to reflect the additional land required for the facility. Since the cost in the source is already calculated for 2022, no adjustment was performed with the CEPCI. The field surface (Surface_Helio_) is expressed in square meters (m^2^).

1. Reference cost of heliostat^7^: $\mathrm{Heli}o_{\mathrm{ref}}$ = 75 USD_2022_/m^2^
2. Land Cost^7^ (+50%): Land_Cost_ = 3.7065 USD_2022_/m^2^
3. Field density = 0.3
4. Uncertainty = 30%

| $\mathrm{Cost}_{\mathrm{Helio}}=\left( \mathrm{Heli}o_{\mathrm{ref}}\cdot\mathrm{Surface}_{\mathrm{Helio}}+\frac{\mathrm{Surface}_{\mathrm{Helio}}}{Field density}\cdot\mathrm{Land}_{\mathrm{Cost}} \right)\cdot\left( 1+MC\cdot Uncertainty \right)$ | Equation 13 |
| --- | --- |

1. **Solar Tower**

The solar tower cost was calculated with a correlation extracted from literature^7^ that considers towers with a single receiver that operate at high temperatures (up to 1000 °C). Since the cost in the source is already calculated for 2022, no adjustment was performed with the CEPCI. The tower height is expressed in meters (m).

1. First correlation constant ^7^: K_1_ = 14.7
2. Correlation exponent^7^: α = 2.64
3. Second correlation constant^7^: K_2_ = 3.91∙10^6^
4. Uncertainty = 30%

| $\mathrm{Cost}_{\mathrm{Tower}}=(K_{1}\cdot{\mathrm{Tower}_{\mathrm{height}}}^{\alpha}+K_{2})\cdot\left( 1+MC\cdot Uncertainty \right)$ | Equation 14 |
| --- | --- |

1. **Solar Calciner**

The cost of the solar calciner is composed of three subsystems: the receiver, the particle lift, and the hot duct and piping. The cost of the receiver depends on its aperture area (expressed in m^2^), while the cost of the particle lift can be obtained with the height of the solar tower and the design flowrate of solids (expressed in m and kg/s, respectively). Finally, the cost of duct and hot piping can be derived from the solar tower height (expressed also in m). Since the cost in the source is already calculated for 2022, no adjustment was performed with the CEPCI.

1. Receiver constant (+10% for high temperature)^7^: K_rec_ = 8.393∙10^4^
2. Lift constant^7^: K_Lift_ = 58.37
3. First piping constant^7^: K_Pipe1_ = 6∙10^3^
4. Second piping constant^7^: K_Pipe2_ = 9∙10^4^
5. Uncertainty = 50%

| ${Cost}_{SCal}=\left( K_{rec}\cdot Aperture+K_{Lift}\cdot\left( Tower_{Height}+10 \right)\cdot Solids_{Flow}+(K_{Pipe_{1}}\cdot Tower_{Height}+K_{Pipe_{2}}) \right)\cdot\left( 1+MC\cdot Uncertainty \right)$ | Equation 15 |
| --- | --- |

1. **Storage**

A correlation is used for the storage units. The correlation is available in the literature as a graph that has been digitalized and fitted to an exponential equation. In order to avoid exceeding the limits of correlation, an upper limit has been set. If reached, a parallel unit is added to reduce the size of the system. This operation is expressed as “Roundup”. To obtain the field cost of each storage unit, the Lang factor of 4 is applied, which accounts for the installation of the equipment on site^5^.

1. Upper limit^6^ = 52217.1 m^3^
2. First constant^6^: K_S1_ = 38.07
3. Second constant^6^: K_S2_ = 97759
4. Void fraction^8^: $\mathrm{voi}d_{\mathrm{frac}}$ = 0.405
5. Uncertainty = 30%
6. **Water**

For the calculation of the water storage, a tank that contains the daily design production capacity of the desalination plant is considered (shown as water_day_ and expressed in m^3^).

1. Units = Roundup ($\mathrm{water}_{\mathrm{day}}$/ Upper limit)
2. Capacity_unit = water_day_ / Units

| ${Cost}_{WS}=\left( K_{S1}\cdot Capacity_{unit}+K_{S2} \right)\cdot Units\cdot\frac{{CEPCI}_{2022}}{{CEPCI}_{2002}}\cdot\left( 1+MC\cdot Uncertainty \right)\cdot Lang\_F$ | Equation 16 |
| --- | --- |

1. **CaCO_3_**

The amount of calcium carbonate to be stored (Capacity_CaCO3_, expressed in kg) is obtained from the model. It considers the minimum size that can accommodate enough CaCO_3_ to meet the required supply of the intermittent section along the daily and seasonal oscillations. The considered density of CaCO3 ($\rho_{\mathrm{CaC}O_{3}}$) is 2710 kg/m^3^.

1. Volume_CaCO3_ = $\frac{\mathrm{Capacity}_{\mathrm{CaC}O_{3}}}{\left( \rho_{\mathrm{CaC}O_{3}}\cdot\left( 1-void_{\mathrm{frac}} \right) \right)}$
2. Units = Roundup ($\mathrm{Volume}_{\mathrm{CaC}O_{3}}$ / Upper limit)
3. Capacity_unit = $\mathrm{Volume}_{\mathrm{CaC}O_{3}}$/ Units

| ${Cost}_{CaCO_{3S}}=\left( K_{S1}\cdot{Capacity}_{unit}+K_{S2} \right)\cdot Units\cdot\frac{{CEPCI}_{2022}}{{CEPCI}_{2002}}\cdot\left( 1+MC\cdot Uncertainty \right)\cdot Lang\_F$ | Equation 17 |
| --- | --- |

1. **CaO**

The amount of calcium oxide to be stored (Capacity_CaO_, expressed in kg) is obtained from the model. It considers the minimum size that can accommodate enough CaO to meet the required supply of the continuous section along the daily and seasonal oscillations. The considered density of CaO ($\rho_{\mathrm{CaO}}$) is 3340 kg/m^3^.

1. Volume_CaO_ = $\frac{\mathrm{Capacity}_{\mathrm{CaO}}}{\left( \rho_{\mathrm{CaO}}\cdot\left( 1-void_{\mathrm{frac}} \right) \right)}$
2. Units = Roundup ($\mathrm{Volume}_{\mathrm{CaO}}$ / Upper limit)
3. Capacity_unit = $\mathrm{Volume}_{\mathrm{CaO}}$/ Units

| $\mathrm{Cost}_{\mathrm{Ca}O_{s}}=\left( K_{S1}\cdot\mathrm{Capacity}_{\mathrm{unit}}+K_{S2} \right)\cdot Units\cdot\frac{\mathrm{CEPCI}_{2022}}{\mathrm{CEPCI}_{2002}}\cdot\left( 1+MC\cdot Uncertainty \right)\cdot Lang\_F$ | Equation 18 |
| --- | --- |

1. **CO_2_**

The amount of CO_2_ stored (shown as Capacity_CO2_ and expressed in kg) is extracted from the model and corresponds to two weeks of production in order to facilitate logistics, compensate for solar irradiation fluctuations and allow for maintenance operations. The considered density of CO_2_ is 743.9 kg/m^3^ (@151 barg and 45 °C).

1. $\mathrm{Volume}_{\mathrm{CO}_{2}}$= $\frac{\mathrm{Capacity}_{\mathrm{CO}_{2}}}{\rho_{\mathrm{CO}_{2}}}$
2. Units = ${Roundup (Volume}_{\mathrm{CO}_{2}}$ / Upper limit)
3. Capacity_unit = $\mathrm{Capacity}_{\mathrm{CO}_{2}}$/ Units

| ${Cost}_{CO_{2s}}=\left( K_{S1}\cdot Capacity\_unit+K_{S2} \right)\cdot Units\cdot\frac{{CEPCI}_{2022}}{{CEPCI}_{2002}}\cdot\left( 1+MC\cdot Uncertainty \right)\cdot Lang\_F$ | Equation 19 |
| --- | --- |

1. **Desalination**

For the desalination plant, a correlation is used that was obtained by digitizing a plot available in the literature^9^. The design value is calculated by the model and corresponds to the daily capacity of the plant on the day with the highest water demand of the whole year (shown as water_day_ and expressed in m^3^).

1. Constant ($K_{\mathrm{desal}}$) = 0.011978
2. Alpha (α) = 0.80732
3. Uncertainty = 30%

| ${Cost}_{Desal}=K_{desal} \cdot{water_{day}}^{\alpha}\cdot{10}^{6}\cdot\frac{{CEPCI}_{2022}}{{CEPCI}_{2008}}\cdot\left( 1+MC\cdot Uncertainty \right)$ | Equation 20 |
| --- | --- |

1. **PV System**
2. **Solar panels and inverter**

The sizing of the solar panels and inverters is obtained from the model and expressed in installed capacity power (showed as PV_inst_ and expressed in W). This value is then multiplied by the specific cost of the solar panels and inverters obtained from the literature^10^.

1. Constant ($K_{\mathrm{pv}}$)^10^ = 0.43546 USD_2019_/W
2. Uncertainty = 30%

| ${Cost}_{PV}=K_{pv} \cdot PV_{inst}\cdot\frac{{CEPCI}_{2022}}{{CEPCI}_{2019}}\cdot\left( 1+MC\cdot Uncertainty \right)$ | Equation 21 |
| --- | --- |

1. **Battery**

The sizing of the battery is obtained from the model and expressed in installed capacity (showed as Bat_inst_ and expressed in Wh). This value is then multiplied by the specific cost of the battery obtained from the literature^10^.

1. Constant ($K_{\mathrm{bat}}$)^10^ = 0.23701 USD_2019_/Wh
2. Uncertainty = 30%

| ${Cost}_{bat}=K_{bat} \cdot{Bat}_{inst}\cdot\frac{{CEPCI}_{2022}}{{CEPCI}_{2019}}\cdot\left( 1+MC\cdot Uncertainty \right)$ | Equation 22 |
| --- | --- |

1. **Operational expenditure (OPEX)**

The variable operational expenditure (OPEX) considers the labor costs to operate the plant and the consumption of the two main chemicals used in the process, calcium carbonate (CaCO_3_) and potassium hydroxide (KOH).

1. **Labor costs**

A correlation is used for the labor costs. The correlation is available in the literature^6^ as a graph that has been digitalized and fitted to an exponential equation. This correlation considers the type of plant, its production capacity (expressed in kg/d) and the number of processing steps. A highly automated plant with large equipment was assumed with a total of three steps, namely continuous section, intermittent section and auxiliary units (solar equipment, storage, desalination and PV plants). The labor wage was assumed to be 25.58 USD_2001_/h^6^, projected from 2001 to 2022 with the United States Nonfarm Unit Labour Costs^11^ and multiplied by 1.15 to include the supervision costs^6^.

1. Constant (K_Labor_) = 2.0488
2. Alpha (α) = 0.2441

| ${Cost}_{Labor}=K_{Labor} \cdot{Production}^{\alpha}\cdot Steps\cdot\left( Wage\cdot\frac{{Unit Labor Cost}_{2022}}{{Unit Labor Cost}_{2001}} \right)\cdot1.15$ | Equation 23 |
| --- | --- |

1. **Chemicals costs**

The amount of CaCO3 required was calculated from the literature^2^ and corresponds to 0.0313 t CaCO_3_/t CO_2_ captured. The quantity of KOH consumed is 0.00290 t KOH/t CO_2_ captured assuming a 99.99% recovery of the chemical^12^. The prices were estimated from online sources^13,14^.

| ${Cost}_{Chemicals}=Consumption_{CaCO_{3}}\cdot Price_{CaCO_{3}}+Consumption_{KOH}\cdot Price_{KOH}$ | Equation 24 |
| --- | --- |

**Table S2.** Summary of the methodology used to calculate the cost of each unit for solar liquid direct air capture (L-DAC). TRL stands for technology readiness level.

| **Category** | **Equipment** | **Basis** | **Estimation method** | **Type of result** | **TRL** | **Lang factor** | **Uncertainty (%)** | **Source** |
| --- | --- | --- | --- | --- | --- | --- | --- | --- |
| Continuous | Air contactor | Nominal hourly airflow | Seven-tenths rule  (K = 1) | Field cost | Low | - | 50% | ^2^ |
| Continuous | Pellet reactor | Hourly solution flowrate | Seven-tenths rule  (K = 0.675) | Field cost | Low | - | 50% | ^2^ |
| Continuous | Filter | Hourly solution flowrate | Seven-tenths rule  (K = 0.625) | Field cost | High | - | 30% | ^2^ |
| Continuous | Steam slaker | CaO hourly flowrate | Seven-tenths rule  (K = 0.675) | Field cost | Low | - | 50% | ^2^ |
| Continuous | Continuous turbine | Nominal power output | Correlation | Purchased cost | High | 4 | 30% | ^6^ |
| Continuous | Other equipment | Cost of continuous units | Linear | Field cost | High | - | - | ^2^ |
| Intermittent | CO_2_ compressor  (4 stages) | Nominal power input | Correlation | Purchased cost | High | 4 | 30% | ^6^ |
| Intermittent | Intermittent turbine | Nominal power output | Correlation | Purchased cost | High | 4 | 30% | ^6^ |
| Intermittent | Other equipment | Cost of intermittent units | Linear | Field cost | High | - | - | ^2^ |
| Solar | Solar calciner | Thermal capacity, tower height and particle flowrate | Correlation | Field cost | Low | - | 50% | ^7^ |
| Solar | Solar tower | Tower height | Linear | Field cost | High | - | 30% | ^7^ |
| Solar | Heliostat field | Field mirror area | Linear | Field cost | High | - | 30% | ^7^ |
| Storage | Water storage | Design capacity | Correlation | Purchased cost | High | 4 | 30% | ^6^ |
| Storage | CaO storage | Design capacity | Correlation | Purchased cost | High | 4 | 30% | ^6^ |
| Storage | CaCO_3_ storage | Design capacity | Correlation | Purchased cost | High | 4 | 30% | ^6^ |
| Storage | CO_2_ storage | Design capacity | Correlation | Purchased cost | High | 4 | 30% | ^6^ |
| Desalination | Whole desalination plant | Daily desalination capacity | Correlation | Field cost | High | - | 30% | ^9^ |
| PV | PV System | Nominal power installed | Linear | Field cost | High | - | 30% | ^10^ |
| PV | Battery | Nominal capacity | Linear | Field cost | High | - | 30% | ^10^ |

## Cost estimation methodology for Conventional L-DAC

The cost estimation of the units found in the conventional L-DAC consisted of adjusting the cost of each unit as reported in the literature^2^ to 2022 using the CEPCI index and to different sizes using the "seven-tenths rule". Similar to the solar L-DAC, a different factor was used for each unit based on the recommendations found in the literature^3^. In the equation below, the reference size used for the conventional L-DAC in the denominator is the annual CO_2_ production (considering the C-Gas/C-Air ratio of 0.48, a design CO_2_ capture capacity of 0.98 Mt CO_2_ and a utilization rate of 90%). A list of the considered units and the respective scaling factors utilized can be observed in Table S3.

Additionally, a range of uncertainty was considered for each unit depending on the TRL (30% for high TRL, 50% for low TRL). This uncertainty is used in the Monte Carlo simulation, which randomly generates a variable (called "MC" in the equations) between -1 and 1.

| ${Cost}_{Unit}=\left( \frac{{CO}_{2} Production \left( \frac{Mt {CO}_{2}}{y} \right)}{0.98\cdot0.9\cdot\left( 1+0.48 \right)} \right)^{Factor}\cdot Cost_{Unit}\cdot\frac{{CEPCI}_{2022}}{{CEPCI}_{2016}}\cdot(1+MC\cdot Uncertainty)$ | Equation 23 |
| --- | --- |

As an exception, the cost of the “other equipment” category was not escalated as described above, but calculated as a fraction of the total cost of the remaining process equipment (excluding the air separation unit). This category includes unlisted units such as heat exchangers, pumps, separators, and mixers, which are assumed to cost 20%^2^ of the process equipment.

| ${Cost}_{Other}=(Cost_{Air contactor}+Cost_{Pellet reactor}+Cost_{Calciner\text{-}slaker}+Cost_{Compressor}+Cost_{Steam turbine}+Cost_{Power plant}+Cost_{Fines filter})\cdot0.2$ | Equation 24 |
| --- | --- |

**Table S3.** List of units for the conventional liquid direct air capture (L-DAC) process, their respective costs, the scaling factor used, and the technology readiness level (TRL) considered for the uncertainty determination.

| **Unit** | **Total direct field costs (MUSD_2016_)^2^** | **Scaling factor utilized^3^** | **TRL** |
| --- | --- | --- | --- |
| Air contactor | 212.2 | 1 | Low |
| Pellet reactor | 130.7 | 0.675 | Low |
| Calciner-slaker | 77.7 | 0.675 | Low |
| Air separation unit | 54.3 | 0.68 | High |
| CO_2_ compressor | 19.9 | 0.4 | High |
| Steam turbine | 7.51 | 0.7 | High |
| Power plant | 35.03 | 0.68 | High |
| Fines filter | 30.9 | 0.625 | High |
| Other equipment | 102.9 | - | - |
| Buildings | 6.7 | 0.61 | High |
| Transformer | 19.8 | 0.7 | High |

**References**

(1) An, K.; Farooqui, A.; McCoy, S. T. The impact of climate on solvent-based direct air capture systems. *Applied Energy* **2022**, *325*, 119895. DOI: 10.1016/j.apenergy.2022.119895.

(2) Keith, D.; Holmes, G.; St. Angelo, D.; Heidel, K. A Process for Capturing CO2 from the Atmosphere. *Joule* **2018**, *2* (1573-1594). DOI: 10.1016/j.joule.2018.05.006.

(3) Remer, D. S.; Chai, L. H. *Process Equipment, Cost Scale-up*; Encyclopedia of Chemical Processing and design, Vol. 43; Marcel Dekker, Inc., 1993.

(4) National Academies of Sciences, Engineering, and Medicine. Negative Emissions Technologies and Reliable Sequestration: A Research Agenda **2019.** DOI: 10.17226/25259.

(5) Physics, A. P. Direct Air Capture of CO2 with Chemicals: A Technology Assessment for the APS Panel on Public Affairs. *APS Physics* **2011**.

(6) Peters, M. S.; Timmerhaus, K. D.; West, R. E. *Plant design and economics for chemical engineers*, 5th ed. / Max S. Peters, Klaus D. Timmerhaus, Ronald West; McGraw-Hill chemical engineering series; McGraw-Hill, 2003.

(7) Buck, R.; Sment, J. Techno-economic analysis of multi-tower solar particle power plants. *Solar Energy* **2023**, *254*, 112–122. DOI: 10.1016/j.solener.2023.02.045.

(8) Seckendorff, J.; Hinrichsen, O. Review on the structure of random packed‐beds. *Can J Chem Eng* **2021**, *99* (S1). DOI: 10.1002/cjce.23959.

(9) Wittholz, M. K.; O'Neill, B. K.; Colby, C. B.; Lewis, D. Estimating the cost of desalination plants using a cost database. *Desalination* **2008**, *229* (1-3), 10–20. DOI: 10.1016/j.desal.2007.07.023.

(10) Eero Vartiainen; Gaëtan Masson; Christian Breyer; David Moser; Eduardo Román Medina. Impact of weighted average cost of capital, capital expenditure, and other parameters on future utility‐scale PV levelised cost of electricity.

(11) U.S. Bureau of Labor Statistics. *Productivity*. https://​www.bls.gov​/​productivity/​tables/​home.htm (accessed 2023-07-24).

(12) Madhu, K.; Pauliuk, S.; Dhathri, S.; Creutzig, F. Understanding environmental trade-offs and resource demand of direct air capture technologies through comparative life-cycle assessment. *Nat Energy* **2021**, *6* (11), 1035–1044. DOI: 10.1038/s41560-021-00922-6.

(13) Qingdao ECHEMI Digital Technology Co., Ltd. *Potassium hydroxide Market Price & Analysis*. https://​www.echemi.com​/​productsInformation/​pd20150901020-​potassium-​hydroxide.html (accessed 2023-07-23).

(14) Qingdao ECHEMI Digital Technology Co., Ltd. *Calcium carbonate Market Price & Analysis*. https://​www.echemi.com​/​productsInformation/​pid_​Seven31057-​calciumcarbonate.html (accessed 2023-07-23).
